# Increased Frequency of T Follicular Helper Cells and Elevated Interleukin-27 Plasma Levels in Patients with Pemphigus

**DOI:** 10.1371/journal.pone.0148919

**Published:** 2016-02-12

**Authors:** Tina Hennerici, Robert Pollmann, Thomas Schmidt, Maria Seipelt, Björn Tackenberg, Christian Möbs, Kamran Ghoreschi, Michael Hertl, Rüdiger Eming

**Affiliations:** 1 Department of Dermatology and Allergology, Philipps University Marburg, Marburg, Germany; 2 Department of Neurology, Philipps University Marburg, Marburg, Germany; 3 Department of Dermatology, Eberhard Karls University Tübingen, Tübingen, Germany; COCHIN INSTITUTE, Institut National de la Santé et de la Recherche Médicale, FRANCE

## Abstract

Pemphigus is an autoimmune disease in which IgG auto-antibodies (auto-ab) against the desmosomal cadherins desmoglein (Dsg) 3 and Dsg1 cause loss of epidermal keratinocyte adhesion. Aim of this study was to investigate cytokines derived from antigen-presenting cells (APC) and their relation to CD4^+^ T cell subpopulations and to the auto-ab response in pemphigus. In this regard, patients with pemphigus were compared to patients with myasthenia gravis (MG), an unrelated auto-ab–mediated autoimmune disease, and healthy controls. In pemphigus and MG, the plasma concentrations of the APC-derived immunomodulatory cytokine IL-27 were highly increased. Strikingly, IL-27 strongly correlated with Dsg-specific IgG auto-ab titers. T helper (Th) 17 cells were augmented in both pemphigus and MG patients while T follicular helper (Tfh) cells, which are essential in providing B cell help, were increased only in pemphigus along with increasing plasma concentrations of IL-21, a cytokine produced by Th17 and Tfh cells. Moreover, we could detect Dsg3-specific autoreactive T cells producing IL-21 upon *ex vivo* stimulation with Dsg3. These findings suggest that IL-27 and IL-21-producing T cells, are involved in the pathogenesis of pemphigus. The further characterization of IL-21-producing T cells and of the role of IL-27 will lead to a more defined understanding of the auto-ab response in pemphigus.

## Introduction

Pemphigus is an antibody (ab)-mediated autoimmune disease in which auto-ab mainly directed against the desmosomal cadherin Desmoglein (Dsg) 3 and Dsg1 cause loss of keratinocyte adhesion in the human skin. This process, called acantholysis, presents clinically with flaccid blisters and erosions of the skin and mucous membranes [1, 2]. Since the precise immunological events resulting in the breakdown of self-tolerance in pemphigus are not yet completely understood, therapeutic options are mainly confined to broad systemic immunosuppression often causing significant side effects and comorbidities [3]. In pemphigus vulgaris (PV), the most common clinical variant of pemphigus, several *in vitro* and *in vivo* studies demonstrated the critical role of Dsg3-specific CD4^+^ T cells in the generation of Dsg3-specific auto-ab [4–9]. Based on the strong prevalence of distinct human leukocyte antigen (HLA) class II alleles in PV, our group recently showed in an HLA-DRB1*04:02–transgenic mouse model of PV that HLA-DRB1*04:02-restricted T cell recognition of human Dsg3 is critical for the induction of pathogenic IgG abs that were capable of inducing intraepidermal loss of adhesion [10].

Autoreactive CD4^+^ T cells are essential for the pathogenesis of several ab-mediated autoimmune diseases by providing help to autoreactive B cells resulting in the production of antigen-specific auto-ab. Beside pemphigus, the chronic autoimmune neuromuscular disease myasthenia gravis (MG), in which auto-ab against components of the neuromuscular junction cause muscle weakness and abnormal fatigue, is dependent on T cells [11]. To date, alterations in several T cell subsets like CD4^+^CD25^+^ Treg and Th17 cells, have been described for pemphigus and MG and are suggested to play a role in the pathogenesis of these diseases [12–14].

Recently, T follicular helper (Tfh) cells have been newly identified to be critically involved in inflammation and B cell activation in autoimmune disease [15, 16]. Tfh cells are specialized in providing help to B cells in germinal centers (GC) and produce high amounts of IL-21 upon activation. Typically, they express the homing receptor CXC-chemokine receptor 5 (CXCR5), defining the localization to B cell follicles within secondary lymphoid tissues [15, 16]. Based on their ability to control the induction of high-affinity humoral immune responses, Tfh cells have been investigated in several autoimmune disorders, such as systemic lupus erythematosus (SLE), rheumatoid arthritis (RA) and MG [17–19], which are all linked to the presence of pathogenic IgG auto-ab. To our knowledge, a potential contribution of Tfh cells to the pathogenesis of pemphigus has not been elucidated.

Cytokines, primarily produced by antigen-presenting cells (APC), play a crucial role during auto-ab response by mediating the function of autoreactive T cells. Hence, monocytes and dendritic cells (DC) have been shown to be critically involved in the pathogenesis of autoimmune diseases, including SLE, type I diabetes, and psoriasis vulgaris [20]. However, the role of disease-promoting cytokines in pemphigus has not yet been fully understood. Interleukin-27 (IL-27) is produced by activated APC and enhanced expression has been found in inflamed tissues [21, 22]. IL-27 has been thoroughly investigated in several autoimmune disorders, such as inflammatory bowel disease, rheumatoid arthritis (RA), experimental autoimmune encephalitis (EAE), psoriasis, and Sjögren’s syndrome (SS) [23–27]. However, the function of IL-27 in the pathogenesis of pemphigus has not yet been characterized.

The aim of this study was to investigate APC-derived cytokines, including IL-27, and their relation to CD4^+^ T cell subsets and to the auto-ab response in pemphigus. Clinically well-defined pemphigus patients and healthy controls (HC) were analyzed. Patients with the neuromuscular disease MG were included as a further unrelated ab-mediated autoimmune disease in order to demarcate the immunological particularities detected in pemphigus patients from those of other autoimmune disorders.

Here, for the first time we can show that in pemphigus significantly elevated IL-27 plasma concentrations strongly correlate with Dsg-specific IgG auto-ab titers. In contrast, there was no correlation of circulating IL-27 concentrations with the anti-acetylcholine receptor (AChR) auto-ab in MG patients. Circulating (c)Tfh cells (defined as CD4^+^CXCR5^+^ T cells) and Th17 cells (IL-17-producing CD4^+^ T cells) were significantly increased in pemphigus along with increasing IL-21 plasma concentrations suggesting an enhanced activation of IL-21-producing T cells in pemphigus. Furthermore, we could identify autoreactive Dsg3-specific T cells secreting IL-21 upon *ex vivo* stimulation with Dsg3. These findings suggest that APC-derived IL-27 and IL-21, produced by Tfh and Th17 cells may be critical in the pathogenesis of pemphigus.

## Materials and Methods

### Patients and healthy controls

Peripheral blood samples (50mL) were taken from each human subject including pemphigus patients, MG patients and HC. An overview on the pemphigus patients (n = 19) and MG patients (n = 12) used for the study is provided in **S1 and S2 Tables**. This study includes 15 patients with PV, 3 with pemphigus foliaceus and 1 atypical pemphigus patient. Pemphigus patients 1–12 were used for the analysis of APC-derived cytokines and Th cells. Since systemic immunosuppressive therapy with prednisolone significantly impacted APC numbers in a dose-dependent manner as shown for myeloid DC (**S1 Fig**), patients with a daily intake of ≥10mg of prednisolone were not included in this subgroup. Pemphigus patients 10–19 were used for the analysis of Tfh cells, IL-21 plasma concentrations and IL-21-ELISpot assay. As we could not detect a distinct influence of the immunosuppressive therapy on Tfh cell numbers, this group included 4 patients with prednisolone treatment >10mg/d. The MG patients suffered from very mild to moderate disease, whereas the pemphigus patients were categorized as active or remittent according to the definition by Murrel et al. [28]. HC were matched according to gender and age and did not display any signs of autoimmune skin inflammation. Each study participant gave written consent before inclusion in the study, which was approved by the Ethics Committee of the Medical Faculty of Philipps-University, Marburg. The study was conducted according to the Declaration of Helsinki Principles.

### Whole-blood flow cytometry

Peripheral blood samples were incubated for 30min at 4°C with the following abs: mouse anti-human CD4 (RPA-T4) -fluorescein isothiocyanate (FITC), mouse anti-human CD11c (B-ly6) -allophycocyanin (APC), rat anti-human CXCR5 (RF8B2) -peridinin chlorophyll protein complex-cyanine5.5 (PerCP5.5; all BD Biosciences, Heidelberg, Germany), anti-human CD14 (MEM-18) -phycoerythrin (PE; ImmunoTools, Friesoythe, Germany) and the respective isotype controls. Red blood cells were lysed twice using ACK lysis buffer (150mM NH_4_Cl + 1mM KHCO_3_ + 0.1mM EDTA) for 5min at room temperature. After two washing steps with buffer (PBS + 1% bovine serum albumin + 0.1% NaN_3_) cells were resuspended in the same buffer for measurement. Flow cytometry analysis was performed using FACSCalibur (BD Biosciences, Heidelberg, Germany) and FlowJo 7.6 single cell analysis software (TreeStar Inc., Ashland, USA).

### Intracellular cytokine staining

Blood peripheral mononuclear cells (PBMC) were isolated from peripheral blood using Ficoll-Hypaque density gradient centrifugation and frozen at -80°C. After thawing, cells were cultured overnight in RPMI-1640 (Capricorn, Ebsdorfergrund, Germany) supplemented with 10% fetal bovine (Biochrom, Berlin, Germany) and afterwards stimulated with 5ng/mL phorbol myristate acetate (PMA; Promega, Fitchburg, USA) and 500ng/mL ionomycin (Calbiochem, Billerica, USA) for 5 hours at 37°C. Cytokine secretion was blocked by the addition of GolgiStop (BD Biosciences, Heidelberg, Germany) according to the manufacturer’s protocol. Subsequently, cells were stained using the following abs: mouse anti-human CD3-PE (UCHT1) and mouse anti-human CD8-FITC (SK1). Usage of this combination was compared to the mouse anti-human CD4-PE (RPA-T4). Intracellular cytokines were detected by incubation with the following abs: mouse anti-human IFN--APC (25723.11), mouse anti-human IL-4-APC (MP4-25D2), rat anti-human IL-10-APC (JES3-19F1) and mouse anti-human IL-17A-AlexaFluor647 (N49-653; all BD Biosciences, Heidelberg, Germany).

### Cytokine ELISA

Cytokines were quantified by ELISA using the following kits: human IL-6/tumor necrosis factor (TNF)-/IL-27 ELISA Ready-SET-Go! (eBioscience, San Diego, USA) according to the manufacturers’ protocols. Cytokine concentrations were derived from a four parameter logistic calibration curve using GraphPad Prism 6.02 (GraphPad Software Inc., La Jolla, USA).

### ELISpot assay

The enzyme-linked immunospot (ELISpot) assay was performed with modifications according to the previously described protocol [29]. Briefly, PBMC were thawed at 37°C and 10^6^cells/mL were cultured in RPMI-1640 (Capricorn, Ebsdorfergrund, Germany) supplemented with 10% pooled human serum, 100U/mL penicillin, 100μg/mL streptomycin and 2mmol/L L-Glutamine (all from PAA Laboratories). PBMC were stimulated with 10μg/mL of recombinant Dsg3 (produced in baculovirus expression vector system as previously described [30]) or recombinant collagen VII as control. For *in vitro* expansion of Dsg3-specific T cells, IL-2 (10U/mL; Roche, Mannheim, Germany) and IL-7 (10ng/mL; Miltenyi Biotech, Bergisch Gladbach, Germany) were added to all wells after 48 hours. On day 8, PBMC were restimulated with 10μg/mL Dsg3 or collagen VII and seeded at 5x10^5^ and 1x10^5^ per well on an ELISpot plate coated with anti-human-IL-21 monoclonal ab. The cells were incubated for 20 hours at 37°C in a humidified atmosphere containing 5%CO_2_.The detection of IL-21-positive spots was performed according to the manufacturer’s protocol (Mabtech AB, Nacka Strand, Sweden). Spots were counted automatically with an ELISpot plate reader (A.EL.VIS, Hannover, Germany) and mean numbers of Dsg3-specific IL-21-positive spots were determined in duplicates after subtracting the spots of the collagen VII-stimulated wells.

### Detection of auto-antibodies

The presence of auto-ab against Dsg1 and Dsg3 in the sera of the pemphigus patients was detected by anti-Dsg1- and anti-Dsg3-ELISA (Euroimmun, Lübeck, Germany). The presence of auto-ab against AChR in the sera of MG patients was determined by radioimmunoassay (ACHRAB-Assay, DLD Diagnostika, Hamburg, Germany). Auto-ab against striated muscle (titin) in the sera of MG patients were detected by indirect immunofluorescence using the Neurology Mosaic 1 testing system (Euroimmun, Lübeck, Germany). All assays were performed according to the manufacturer’s protocol.

### Statistical analysis

Statistical analysis was performed using GraphPad Prism 6.02 (GraphPad Software Inc., La Jolla, USA). Cumulative data are displayed as scatter plots with median. Comparisons between the groups were carried out using the two-tailed Mann-Whitney-U-Test. Correlation analysis was carried out using two-tailed Spearman’s rank correlation. Differences between the groups were considered as statistically significant at *p* values of <0.05.

## Results

### Elevated plasma levels of IL-27 in pemphigus

**Fig 1:**
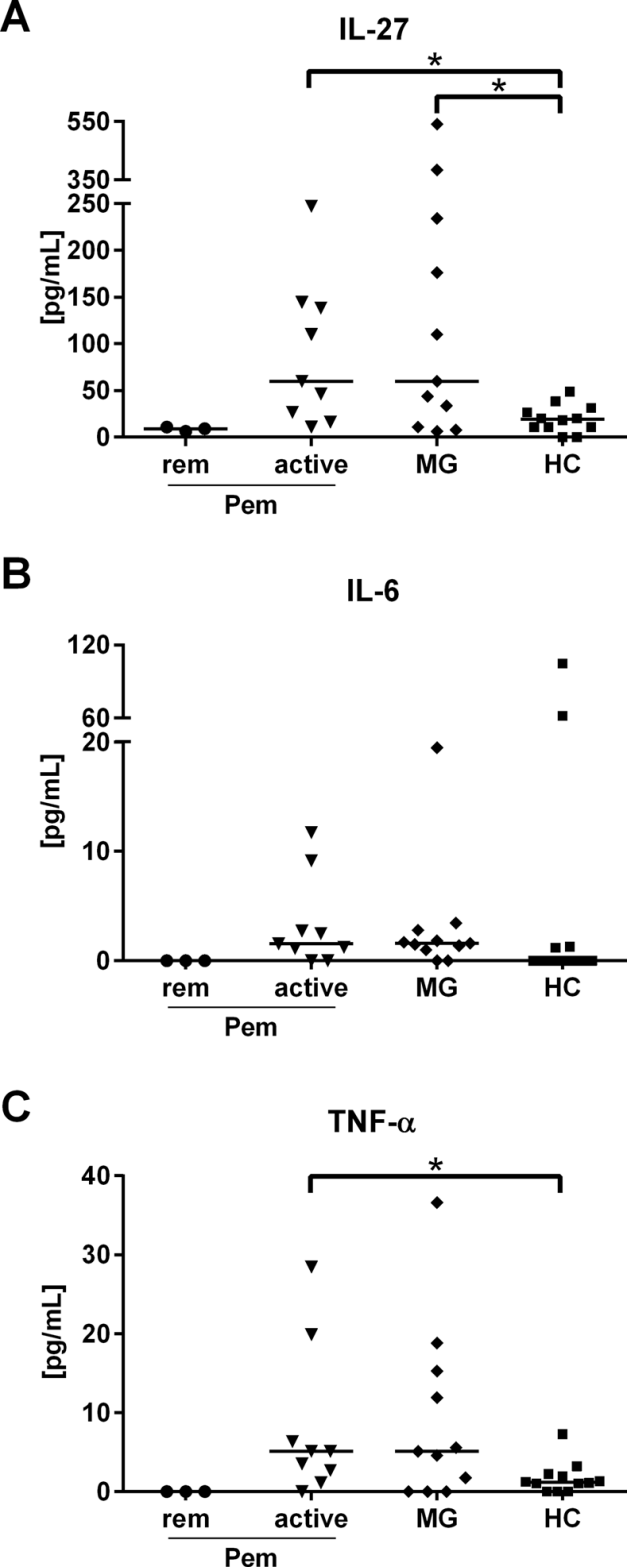
Increased plasma levels of IL-27 in active pemphigus and MG patients. Plasma samples of pemphigus (Pem) patients (n = 12) categorized as active and remittent (rem), MG patients (n = 12) and HC (n = 12) were tested for different cytokines by ELISA. **(A)** Both active Pem and MG patients had significantly increased plasma concentrations of IL-27. **(B)** The plasma levels of IL-6 were not increased in active Pem while **(C)** TNF-α was statistically increased in active pemphigus. Differences between the groups were analyzed using Mann-Whitney-U-Test with *p* values indicated as *p<0.05.

We investigated the plasma concentrations of the APC-derived immunomodulatory cytokine IL-27 and the pro-inflammatory cytokines IL-6 and TNF-α in active and remitting pemphigus patients, MG patients and HC. Strikingly, IL-27 was significantly elevated in active pemphigus and MG (**Fig 1A**). Furthermore, the plasma concentrations of TNF-α were also increased in active pemphigus (**Fig 1C**) and correlated very strongly with the plasma concentrations of IL-27 (*r*_*s*_ = 0.87) (**Table 1**) supporting a pro-inflammatory function of IL-27 in pemphigus. The plasma levels of IL-6 were not increased in active pemphigus and MG, respectively (**Fig 1B**). Interestingly, in the remitting pemphigus patients (*n* = 3) neither IL-27 nor IL-6 or TNF-α could be detected in distinct amounts (**Fig 1A–1C**).

**Table 1:** Correlation of plasma cytokine levels in patients with active pemphigus and MG.

Pem			
	IL-6	TNF-α	IL-27
IL-6	-	-	-
TNF-α	0.092	-	-
IL-27	0.285	**0.870**	-
MG			
	IL-6	TNF-α	IL-27
IL-6	-	-	-
TNF-α	0.583	-	-
IL-27	0.360	**0.936**	-

Spearman’s rank correlation coefficients for a very strong correlation of r_s_ >0.7 are marked in bold.

### Increased IL-10-producing T cells in pemphigus

**Fig 2:**
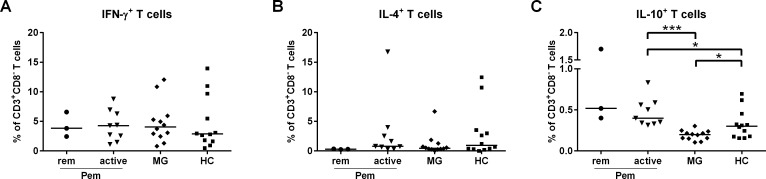
Increased IL-10-producing T cells in active pemphigus. PBMC of pemphigus (Pem) patients (n = 12) categorized as active and remittent (rem), MG patients (n = 12) and HC (n = 12) were used for flow cytometry analysis of the intracellular produced T cell signature cytokines IFN-γ, IL-4 and IL-10 upon stimulation with PMA and ionomycin. In order to circumvent difficulties with stimulation-dependent reduced CD4 expression, CD4^+^ T cells were defined as CD3^+^CD8^-^ cells instead. **(A-B)** IFN-γ-producing T cells and IL-4-producing T cells cell populations did not vary between all groups. **(C)** In active Pem, IL-10-producing T cells were statistically increased, while in MG their frequency was reduced. Differences between the groups were analyzed using Mann-Whitney-U-Test with *p* values indicated as *p<0.05 and ***p0.001.

Considering the observed increased production of APC-derived pro-inflammatory cytokines in plasma of pemphigus patients, we next sought to investigate whether the polarization of certain CD4^+^ T cells is affected by analyzing the production of T cell signature cytokines (IFN-γ, IL-4 and IL-10) with flow cytometry (Gating: **S3 Fig**). We could not see a general shift towards a more pronounced IFN-γ or IL-4 production by T cells, (**Fig 2A and 2B**), but we observed an increased population of IL-10-producing CD4^+^ T cells in pemphigus patients upon stimulation with PMA and ionomycin (**Fig 2C**). Interestingly, in contrast to pemphigus patients IL-10-producing CD4^+^ T cells were significantly reduced in MG compared to HC (**Fig 2D**).

### IL-27 plasma levels strongly correlate with Dsg-specific IgG auto-ab titers in pemphigus

**Fig 3:**
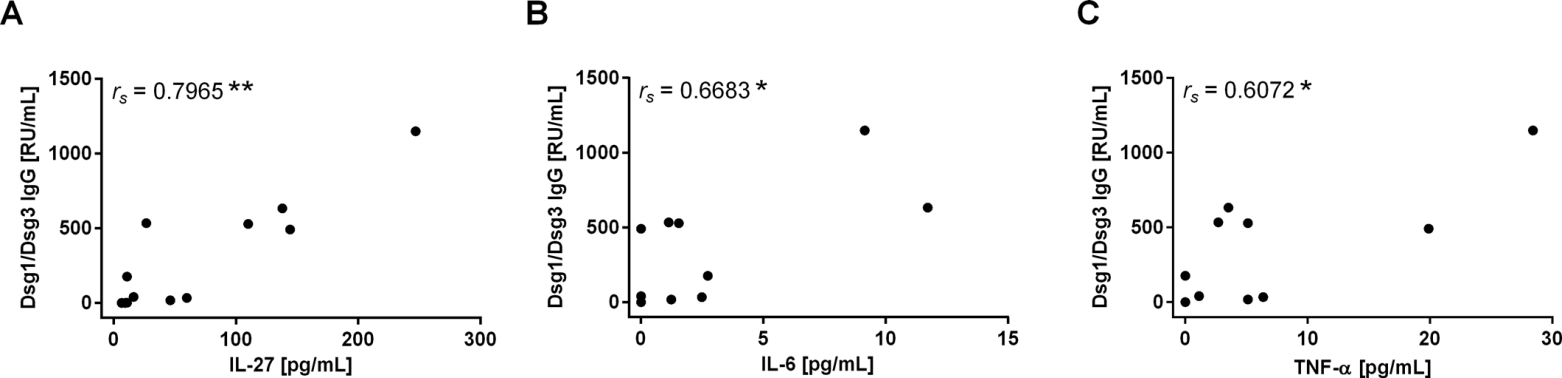
Correlation of Dsg-specific IgG auto-ab titers with plasma levels of IL-27, IL-6, and TNF-α in pemphigus. **(A)** A very strong positive correlation between Dsg1/3 IgG titers and the plasma concentration of IL-27 was found in pemphigus patients (n = 12). **(B-C)** Dsg1/3 IgG titers correlated with the plasma concentrations of the pro-inflammatory cytokine IL-6 and TNF-α in pemphigus. Spearman’s rank correlation coefficients (*r*_*s*_) are shown with *p* values indicated as *p<0.05 and **p0.01.

Our data demonstrated increasing plasma concentrations of APC-derived pro-inflammatory cytokines, especially IL-27, in active pemphigus and MG (**Fig 1A**). To test whether the observed increased cytokine levels can be linked to titers of Dsg-specific IgG auto-ab, correlation analysis of the cytokines IL-27, IL-6, TNF-α and the anti-Dsg1/3 IgG titers was performed. Interestingly, the plasma concentrations of IL-27 showed a very strong correlation with the anti-Dsg1/3 IgG titers (*r*_*s*_ = 0.80) (**Fig 3A**) while a strong correlation could be observed for IL-6 (*r*_*s*_ = 0.67) and TNF-α (*r*_*s*_ = 0.61), respectively (**Fig 3B and 3C**). To further investigate whether these correlations can also be attributed to MG, we also checked for a correlation of cytokine levels with the respective anti-AChR IgG titers in MG patients. In contrast to pemphigus, no correlation between the investigated cytokines and the anti-AChR IgG titers could be observed in MG (**S2 Fig**). The very strong correlation of IL-27 with the anti-Dsg3 IgG titers implicates a disease-specific function of IL-27 in the production of auto-ab in pemphigus.

### Circulating CD4^+^ CXCR5^+^ T cells are increased in pemphigus

**Fig 4:**
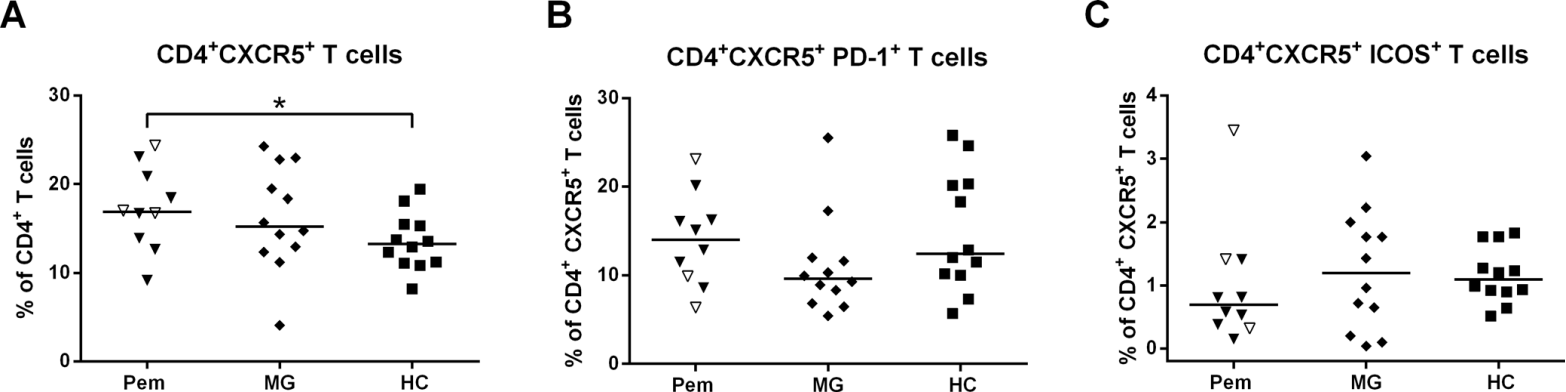
Elevated Tfh cells in pemphigus patients. **(A)** cTfh cells (defined as CD4^+^CXCR5^+^ T cells) were analyzed by flow cytometry in patients with pemphigus (Pem; n = 10), MG (n = 12) and HC (n = 12). (**A**) The frequency of cTfh cells was significantly increased in Pem patients while altered PD-1 (**B**) or ICOS (**C**) expression on cTfh cells could not be observed. Pem patients are categorized as remittent (open symbols) and active (filled symbols). Differences between the groups were analyzed using Mann-Whitney-U-Test with *p* values indicated as *p<0.05.

Following the strong correlation of the IL-27 concentrations with anti-Dsg3 IgG titers, we next addressed the question how the auto-ab production in pemphigus could be mediated by IL-27. Recently, it has been shown that IL-27 can promote T cell-dependent ab responses by enhancing the expansion of Tfh cells which provide essential costimulation to B cells during the development into ab-producing plasma cells in GC [31]. Therefore we analyzed the frequencies of cTfh (defined as CD4^+^CXCR5^+^ T cells as previously described [32]) in the peripheral blood of pemphigus patients with flow cytometry (Gating: **S4 Fig**). cTfh cells were significantly elevated (*p* = 0.04) in pemphigus patients while no statistical difference could be observed in MG patients (**Fig 4A**). CD4^+^CXCR5^+^ cTfh cells were further characterized by analyzing the Tfh markers programmed death-1 (PD-1) and inducible T cell costimulator (ICOS). Pemphigus patients and MG patients showed no difference in the frequencies of PD-1^+^ and ICOS^+^ cTfh cells compared to HC (**Fig 4B and 4C**).

### Elevated IL-21 plasma levels and increased IL-17-producing T cells in pemphigus

**Fig 5:**
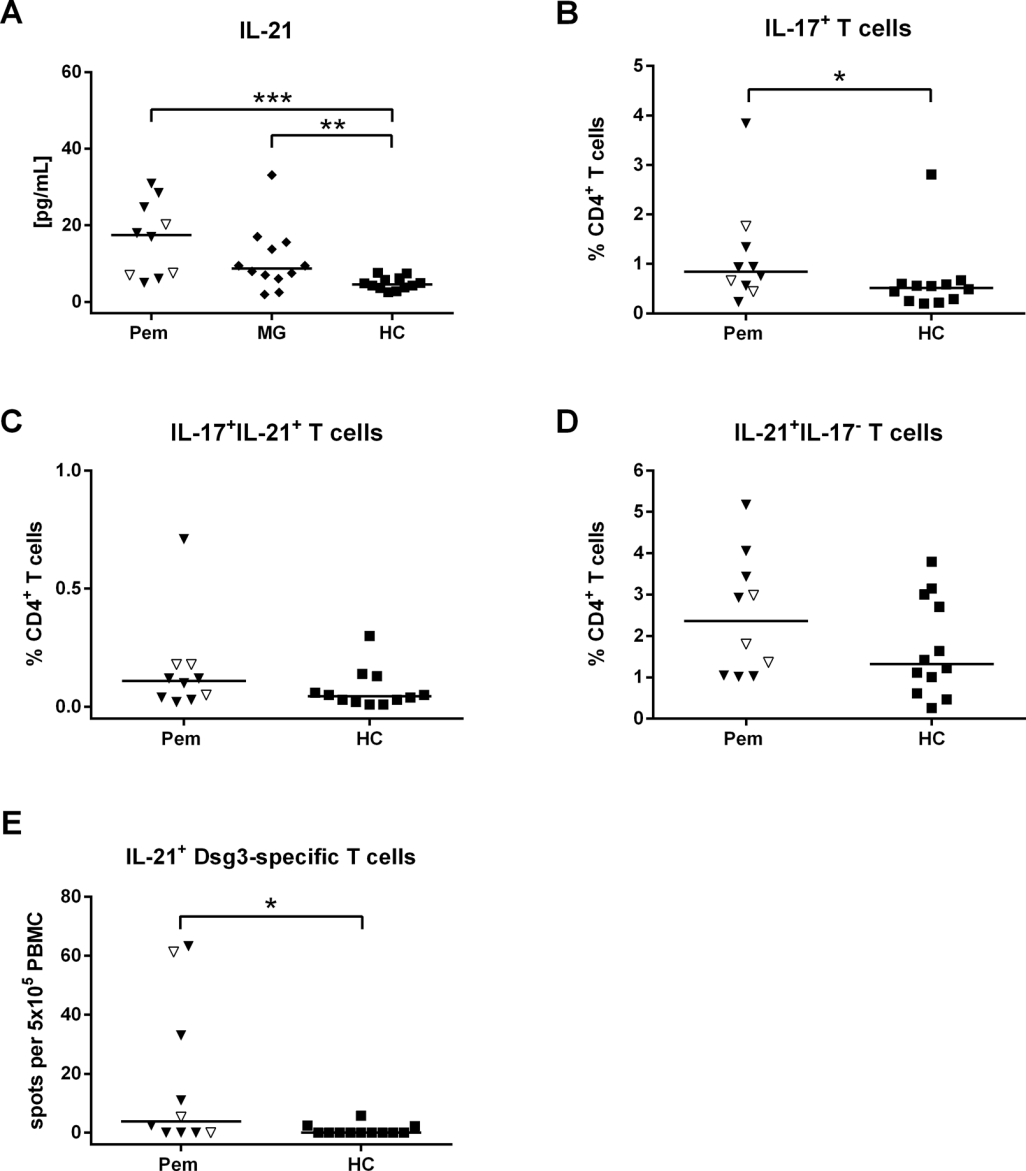
Elevated IL-21 plasma levels and increased IL-17-producing T cells in pemphigus. Patients with pemphigus (Pem; n = 10), MG (n = 12) and HC (n = 12) were analysed for IL-21 and IL-17 (**A**) Active Pem patients as well as MG patients displayed significantly increased plasma concentrations of IL-21. (**B**) An increasing frequency of IL-17-producing T cells could be obersevd in Pem patients while (**C**) IL-17/IL-21 double positive T cells and (**D**) IL-21 single positive T cells showed no statistical difference compared to HC. (**E**) Pem patients showed increased numbers of autoreactive IL-21-producing T cells by ELISpot assay. PBMC were used for flow cytometry analysis of the intracellular produced cytokines upon stimulation with PMA and ionomycin. Pem patients are categorized as remittent (open symbols) and active (filled symbols). Differences between the groups were analyzed using Mann-Whitney-U-Test with *p* values indicated as *p<0.05, **p0.01 and ***p0.001.

As cTfh cells were increased in pemphigus we analyzed IL-21 production in pemphigus patients. IL-21 is a pleiotropic cytokine that is predominantly produced by Tfh and Th17 cells with differential effects on T and B cells including the promotion of B cell proliferation and antibody production [33]. As we found significantly elevated plasma concentrations of IL-21 (*p* = 0.01) in pemphigus patients (**Fig 5A**) we aimed to further track down the cellular source of IL-21. Thus, we analyzed IL-21 and IL-17 production in T cells after *in vitro* stimulation with PMA and ionomycin by flow cytometry (Gating: **S5 Fig**). We observed a significant increase (*p* = 0.04) of IL-17-producing CD4^+^ T cells (**Fig 5B**) while IL-17/IL-21 double positive CD4^+^ T cells showed no difference between pemphigus patients and HC (**Fig 5C**). Of note, IL-21 single positive CD4+ T cells tended to be increased in pemphigus (**Fig 5D**) and could be found more frequently (median: 2.36% CD4^+^ T cells) compared to IL-17/IL-21 double positive CD4+ T cells (median: 0.11% CD4^+^ T cells) in pemphigus patients. Next, we were interested whether Dsg3-specific IL-21-secreting T cells would contribute to the observed elevated IL-21 plasma levels in pemphigus. Therefore we stimulated PBMC from pemphigus patients and HC with Dsg3 protein *ex vivo* and analyzed the number of IL-21-secreting cells by ELISpot assay. After stimulation with Dsg3 we could identify autoreactive IL-21-secreting cells in 50% of the pemphigus patients (**Fig 5E**). These results show for the first time the presence of IL-21^+^ Dsg3-specific T cells in peripheral blood of pemphigus patients.

## Discussion

Cytokines play a crucial role in the pathogenesis of pemphigus by mediating T cell function [34]. However, the role of disease-promoting cytokines in pemphigus has not yet been fully understood. In this study we characterized distinct APC-related cytokines, T cell subpopulations and their correlation with Dsg-specific auto-ab in pemphigus patients. Here we show that IL-27 plasma concentrations are increased in pemphigus and in MG, an unrelated auto-ab mediated neurological autoimmune disease (**Fig 1A**). However, IL-27 plasma concentrations strongly correlated with the IgG auto-ab titers only in pemphigus (**Fig 3A**) suggesting a disease-specific function of IL-27 in the pathogenesis of pemphigus. IL-27, a heterodimeric cytokine is evolutionary related to IL-12 and it is expressed mainly by activated APC [35]. The pro-inflammatory capacity of IL-27 has been described in several autoimmune disorders, such as RA [24, 36, 37], EAE [22, 25], psoriasis [26, 38], and SS [27]. For instance, Goldberg et al. suppressed an ongoing disease in mouse models of both RA and multiple sclerosis via the neutralization of the p28 subunit of IL-27 [24, 25]. In our study we noticed a strong correlation of IL-27 plasma concentrations with TNF-α in pemphigus patients which might support an additional pro-inflammatory function of IL-27 in pemphigus as well (**Table 1**). Recently therapeutic targeting of TNF-α alone was shown not to be effective in PV [39], thus the blockade of novel cytokines potentially involved in PV pathogenesis, such as IL-27, may represent a promising treatment option.

Recently, Batten et al. demonstrated that IL-27 signaling in T cells resulted in the production of IL-21 and in addition that IL-27 was able to enhance both the survival of activated CD4^+^ T cells and the expression of the Tfh cell markers CXCR5, PD-1 and ICOS. Moreover, loss of IL-27 signaling was shown to ameliorate disease in a murine model of SLE [31]. Tfh cells represent specialized and essential providers of B cell help both to form and maintain GC and to regulate the maturation of B cells into memory B cells and plasma cells secreting high-affinity abs [16]. Circulating counterparts of Tfh cells can be detected by the expression of CXCR5 in peripheral blood [16, 32]. Alterations in the cTfh compartment have been found in several ab-mediated autoimmune diseases, like SLE and RA, in which the expansion of cTfh cells (defined as CD4^+^CXCR5^+^PD-1^+^ICOS^+^ T cells) could be linked to disease activity [17, 18]. Furthermore, Gringhuis et al. demonstrated that IL-27 produced by activated DC can induce Tfh cell generation and T cell-dependent IgG production by B cells [40]. Since the frequency of CD4^+^CXCR5^+^ cTfh cells was significantly increased in pemphigus patients (**Fig 4A**) the above mentioned axis of IL-27-induced promotion of Tfh cells may also be relevant in pemphigus although a correlation between cTfh cells and IL-27 could not be observed (**S6 Fig**). The effect of IL-27 on promoting cTfh frequencies might occur in a timely delayed manner that would not be apparent in our cross-sectional study. A subpopulation of cTfh cells also expresses the surface markers PD-1 and ICOS that are essential for Tfh function [15]. Nevertheless, we did not observe increasing frequencies of PD-1^+^ or ICOS^+^ cTfh cells as it has been previously observed in ab-mediated autoimmunity [17, 18, 41] suggesting that in pemphigus the relative number of total cTfh is altered. In contrast to previous studies in MG [19, 41, 42], we did not see an increased frequency of cTfh cells in our cohort of MG patients which might be explained by the rather mild to moderate disease activity of the study patients. In addition, we cannot exclude a potential therapeutic effect since 11 of the 12 MG patients received an immunomodulatory or immunosuppressive therapy at the time of investigation (**S2 Table**). Still, cTfh cells tended to be increased in MG (*p* = 0.13) and patients with a higher frequency of cTfh cells showed AChR-specific IgG while all AChR-seronegative patients had lower cTfh cells (data not shown).

In this study, pemphigus and MG patients showed elevated plasma concentrations of IL-21 (**Fig 5A**), a cytokine which is essential for B cell differentiation into class-switched plasma cells [43]. Increasing IL-21 levels in blood have been previously observed in MG and other auto-ab-mediated autoimmune diseases, such as RA and SLE [44–47] suggesting that IL-21 is produced by autoreactive Tfh cells during an ongoing auto-ab response. Recently, IL-21 levels and Tfh cells were also found to be increased in the pemphigus-related blistering disease bullous pemphigoid [48]. So far, to our knowledge elevated IL-21 levels have not been described in pemphigus, yet. Moreover, we identified Dsg3-specific IL-21-producing cells in a significantly higher number in the analyzed pemphigus patients compared to HC by ELISpot assay (**Fig 5B**). These results fuel the hypothesis that autoreactive Dsg3-specific IL-21-producing T cells contribute to the generation of Dsg3-specific auto-ab by providing help to autoreactive B cells. We identified increased frequencies of Th17 cells that are predominant producers of IL-21 as well. Th17 cells were also studied by Xu et al. who demonstrated significantly elevated numbers of circulating Th17 cells in acute onset and chronic active PV [12]. Recently, Asothai et al. described increased frequencies of Th17 cells in the blood of PV patients as well [13]. In this context we conclude from our present results that IL-21 in pemphigus is partially released by activated Th17 cells since we detected IL-17/IL-21 double positive T cells in our patients (**Fig 5C**) and additionally IL-21 has been shown to promote the differentiation of Th17, as an autocrine factor [49, 50]. However, IL-21-producing Th17 cells only display a minor T cell population in the investigated pemphigus patients (0.11% CD4^+^ T cells) with IL-21 single positive T cells being more frequent (2.36% CD4^+^ T cells). Most likely, these cells represent a population of IL-21-positive Tfh cells although in our pemphigus cohort the median percentage of IL-21 single positive T cells is lower compared to the median frequency of CD4^+^CXCR5^+^ T cells (16.91% CD4^+^ T cells). As we detected intracellular IL-21 in patients’ PBMC after unspecific stimulation with PMA and ionomycin *in vitro* these conditions may not be sufficient to induce IL-21 production in all Tfh cells. However, whether IL-21 single positive T cells are Tfh cells or may belong to another CD4^+^ T cell population requires further investigation. For instance, it has been shown that IL-21 can also be produced by natural killer T cells, which partly express CD4 [51].

A limitation of this study is that 8 pemphigus patients were under treatment receiving corticosteroids and other immunosuppressives (**S1 Table**). It is known that corticosteroids, like prednisolone, can exert different effects on various components of the immune system [52]. As the therapy with prednisolone significantly impacted APC numbers in a dose-dependent manner (**S1 Fig**) pemphigus patients with a prednisolone therapy of >10mg/d were not included in the analysis of the APC-derived IL-27 and Dsg-specific IgG. However, a few pemphigus patients used for analysis of cTfh cells and IL-21 plasma levels were under systemic treatment. Recently, it has been shown for other ab-mediated diseases that immunosuppressive treatment led to a reduction of cTfh cells and IL-21 in the blood [53, 54]. As we observed an increase instead of a reduction of cTfh cells in the analyzed pemphigus patients it is likely that this observation rather reflects a disease-related characteristic in pemphigus.

Remarkably, active pemphigus patients exhibited slightly augmented subpopulations of IL-10-producing CD4^+^ T cells unlike the MG patients who showed reduced percentages of these cells compared to HC (**Fig 2C**). Previous studies demonstrated reduced immunoregulatory CD4^+^ T cell numbers in PV patients, such as IL-10-secreting Dsg3-specific type 1 regulatory T cells [12, 13, 55–57]. In this study we did not further characterize these IL-10 positive CD4+ T cells with regard to their potentially immunosuppressive function. So we cannot fully exclude that the increased number of IL-10-producing CD4^+^ T cells in pemphigus patients may at least partly represent a population of type 1 regulatory T cells that are in transition to Th17 cells as it has been already shown in other inflammatory skin diseases [58].

To summarize, a strong correlation between IL-27 plasma levels and Dsg-specific IgG auto-ab titers, as well as elevated plasma levels of IL-21 along with increased frequencies of cTfh and Th17 cells suggest a potential implication of IL-27 and IL-21-producing T cells in the pathogenesis of pemphigus. Thus, antagonizing IL-27 and IL-21-production may represent a novel therapeutic option in pemphigus.

## Supporting Information

S1 Fig Impact of treatment with prednisolone on circulating DC numbers. **(A-B)** Pemphigus (Pem; n = 34) and MG patients (n = 31) were grouped according to the daily dosage [mg/d] of prednisolone administrated. mDC (CD14^-^CD11c^++^) numbers of both Pem and MG patients significantly diminished with rising doses of prednisolone. Differences between the groups were analyzed using Mann-Whitney-U-Test with *p* values indicated as *p<0.05, **p 0.01 and ***p 0.001.
(TIF)

S2 Fig No correlation of AChR-specific IgG auto-ab titers with plasma levels of IL-27, IL-6 and TNF-α in MG. **(A)** Serum anti-AChR IgG titers did not correlate with plasma IL-27 in MG (n = 12). **(B)** Auto-ab titers in MG did not correlate with the plasma levels of the pro-inflammatory cytokine IL-6 **(C)** A negative correlation between TNF-α and the auto-ab titers was found in MG. Spearman’s rank correlation coefficients (*r*_*s*_) are shown with *p* values indicated as *p<0.05.
(TIF)

S3 Fig Gating strategy for intracellular cytokine detection in Th cells. PBMC stimulated with PMA, ionomycin and monensin were used for the analysis **(A)** Lymphocytes were gated on size and granularity. Th cells were defined as CD3^+^CD8^-^ T cells and intracellular cytokine expression was detected. **(B)** Gates for cytokine detection were defined according to the respective isotype control.
(TIF)

S4 Fig Gating strategy for flow cytometric analysis of CD4^+^CXCR5^+^ cTfh cells. ACK-lysed blood cells were used for analysis. **(A)** Lymphocytes were gated on size and granularity. cTfh cells were identified as CD4^+^ T cells expressing CXCR5 and further characterized by measuring determining PD-1 and ICOS expression. **(B)** Gates were defined according to the respective isotype controls.
(TIF)

S5 Fig Gating strategy for intracellular cytokine detection in CD4^+^ T cells. PBMC stimulated with PMA, ionomycin and monensin were used for the analysis **(A)** Lymphocytes were gated on size and granularity. T cells were gated on CD4 expression and intracellular IL-21 and IL-17 expression was detected. **(B)** The CD4-gate was defined according to the respective isotype control. Gates for IL-21 and IL-17 were defined by staining non-stimulated PBMC with the IL-21 or IL-17 antibody.
(TIF)

S6 Fig No correlation of IL-27 with cTfh frequencies and IL-21. (**A**) IL-27 plasma levels do not correlate with CD4^+^CXCR5^+^ T cells **(B)** and IL-21 plasma levels. Spearman’s rank correlation coefficients (*r*_*s*_) are shown.
(TIF)

S1 Table Clinical phenotype and auto-ab profile of patients with pemphigus. (DOCX)

S2 Table Clinical status and auto-ab profile of patients with myasthenia gravis. (DOCX)

## Abbreviations

AChR: acetylcholine receptor
ab: antibody
APC: antigen-presenting cell
auto-ab: auto-antibody
c: circulating
CXCR5: CXC-chemokine receptor 5
DC: dendritic cells
Dsg: desmoglein
GC: germinal center
HC: healthy control
MG: myasthenia gravis
PBMC: peripheral blood mononuclear cells
PV: pemphigus vulgaris
RA: rheumatoid arthritis
SLE: systemic lupus erythematosus
Tfh: T follicular helper
TNF: tumor necrosis factor
