# Supplementary figures and images for: Increased Frequency of T Follicular Helper Cells and Elevated Interleukin-27 Plasma Levels in Patients with Pemphigus
Source: PLoS One. 2016 Feb 12;11(2):e0148919. doi: 10.1371/journal.pone.0148919 (PMC4752242; doi:10.1371/journal.pone.0148919)

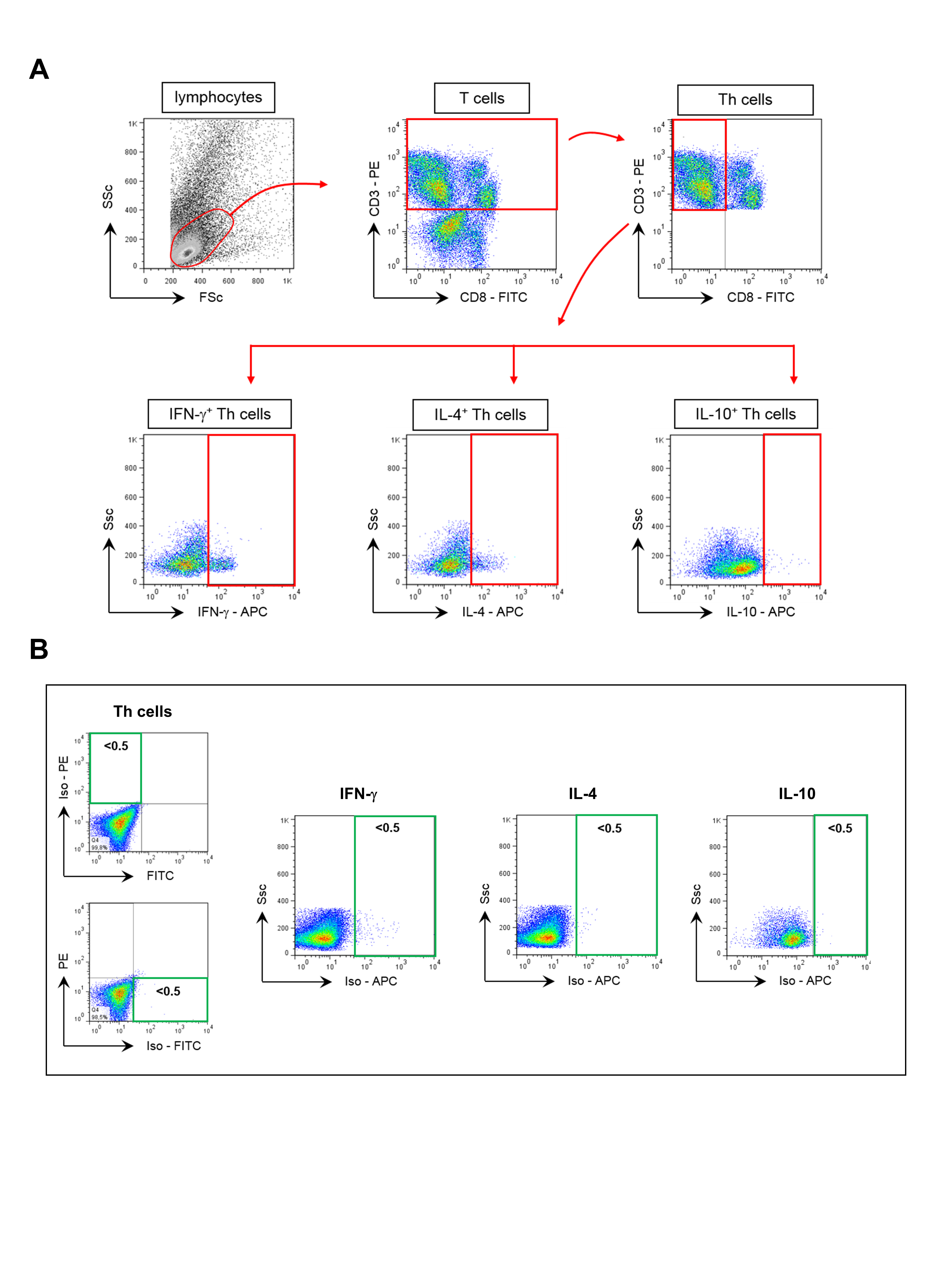

Supplement: S3 Fig — PBMC stimulated with PMA, ionomycin and monensin were used for the analysis (A) Lymphocytes were gated on size and granularity. Th cells were defined as CD3+CD8- T cells and intracellular cytokine expression was detected. (B) Gates for cytokine detection were defined according to the respective isotype control. (TIF) [file pone.0148919.s003.tif]

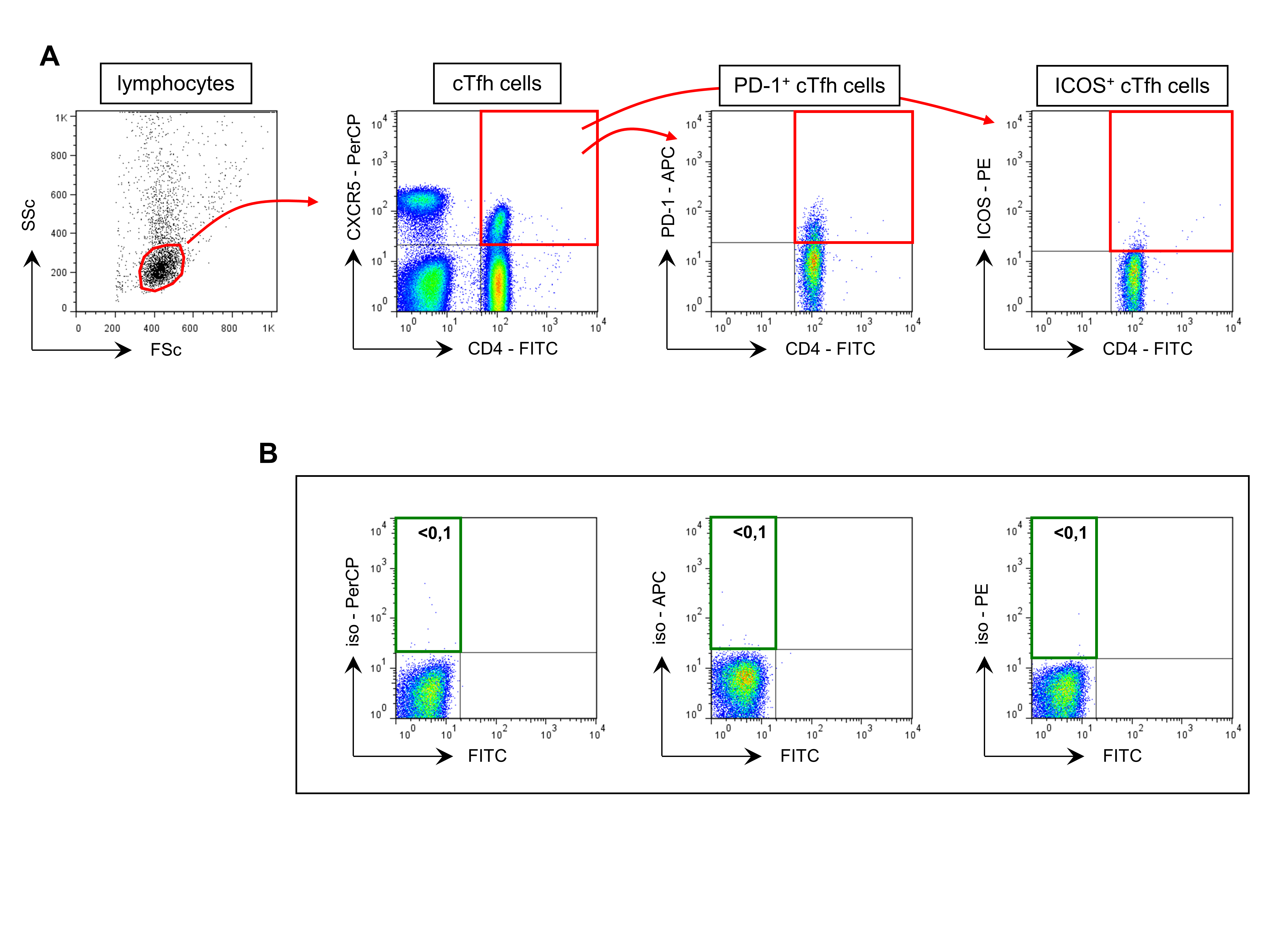

Supplement: S4 Fig — ACK-lysed blood cells were used for analysis. (A) Lymphocytes were gated on size and granularity. cTfh cells were identified as CD4+ T cells expressing CXCR5 and further characterized by measuring determining PD-1 and ICOS expression. (B) Gates were defined according to the respective isotype controls. (TIF) [file pone.0148919.s004.TIF]

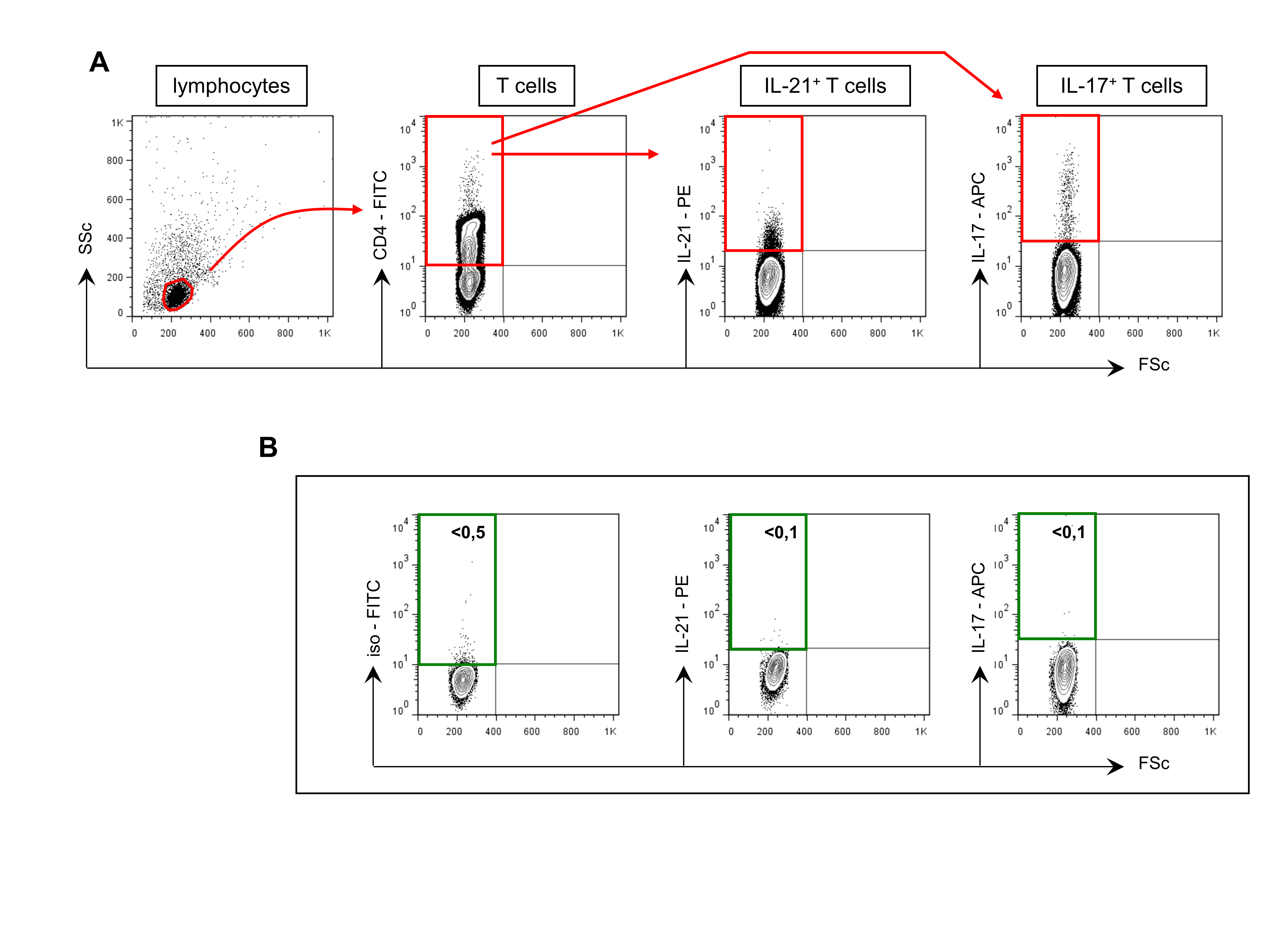

Supplement: S5 Fig — PBMC stimulated with PMA, ionomycin and monensin were used for the analysis (A) Lymphocytes were gated on size and granularity. T cells were gated on CD4 expression and intracellular IL-21 and IL-17 expression was detected. (B) The CD4-gate was defined according to the respective isotype control. Gates for IL-21 and IL-17 were defined by staining non-stimulated PBMC with the IL-21 or IL-17 antibody. (TIF) [file pone.0148919.s005.TIF]
