# Supplementary material for: Increased Frequency of T Follicular Helper Cells and Elevated Interleukin-27 Plasma Levels in Patients with Pemphigus
Source: PLoS One. 2016 Feb 12;11(2):e0148919. doi: 10.1371/journal.pone.0148919 (PMC4752242; doi:10.1371/journal.pone.0148919)
Supplement: S1 Table — (DOCX) [file pone.0148919.s007.docx]

S1 Table. Clinical phenotype and auto-ab profile of patients with pemphigus.

|  |  |  | Clinical phenotype | |  |  | Auto-ab (IgG)^3^ | |
| --- | --- | --- | --- | --- | --- | --- | --- | --- |
| Patient | Type | Status^1^ | Skin | Mucosa |  | Medication^2^ | Dsg3 | Dsg1 |
|  |  |  |  |  |  |  |  |  |
| 1 | P. foliaceus | Active | head and trunk | - |  | None | - | 34 |
| 2 | P. vulgaris | Partial Remission  (off therapy) | - | oral |  | None | - | - |
| 3 | P. vulgaris | Active | head, trunk, legs | - |  | 2g MMF^4^ | - | 632 |
| 4 | P. vulgaris | Complete Remission  (off therapy) | - | - |  | None | - | - |
| 5 | atypical P. | Active | legs and feet | oral |  | None | - | - |
| 6 | P. vulgaris | Active | - | oral |  | None | 40 | - |
| 7 | P. vulgaris | Active | - | oral |  | None | 176 | - |
| 8 | P. folicaceus | Active | head and trunk | - |  | None | - | 534 |
| 9 | P. vulgaris | Complete Remission  (off therapy) | - | - |  | None | - | - |
| 10 | P. vulgaris | Active | - | genital |  | None | 1148 | - |
| 11 | P. vulgaris | Active | - | oral |  | None | 529 | - |
| 12 | P. vulgaris | Active | - | oral |  | None | 491 | - |
| 13 | P. foliaceus | Complete Remission  (on therapy) | - | - |  | 15mg Pred^5^  1g MMF | - | - |
| 14 | P. vulgaris | Partial Remission  (on therapy) | head | - |  | 3.75mg Pred | 135 | - |
| 15 | P. vulgaris | Active | head and trunk | - |  | 40mg Pred  200mg AZA^6^ | 422 | 143 |
| 16 | P. vulgaris | Active | trunk and extremities | oral |  | 10mg Pred  2g MMF | 1759 | 1556 |
| 17 | P. vulgaris | Partial Remission  (on therapy) | breast | - |  | 10mg Pred | 175 | 340 |
| 18 | P. vulgaris | Active | head, trunk, extremities | oral |  | 40mg Pred | 75 | 1582 |
| 19 | P. vulgaris | Active | head, trunk, extremities | oral |  | 30mg Pred  150mg AZA | 330 | 145 |

^1^ as defined by Murrell et al. 2008

^2^ daily medication at time of study

^3^ as determined by ELISA with recombinant Dsg1 or Dsg3 in RU/mL. The cut-off value is 20 RU/mL

^4^ mycophenolate mofetil

^5^ prednisolone

^6^ azathioprine
