# Supplementary material for: Increased Frequency of T Follicular Helper Cells and Elevated Interleukin-27 Plasma Levels in Patients with Pemphigus
Source: PLoS One. 2016 Feb 12;11(2):e0148919. doi: 10.1371/journal.pone.0148919 (PMC4752242; doi:10.1371/journal.pone.0148919)
Supplement: S2 Table — (DOCX) [file pone.0148919.s008.docx]

S2 Table. Clinical status and auto-ab profile of patients with myasthenia gravis

|  |  |  | Auto-ab profile (IgG)^2^ | |
| --- | --- | --- | --- | --- |
| Patient | Status | Medication^1^ | AChR | Titin |
|  |  |  |  |  |
| 1 | Very mild | IVIg^3^ | 1.6 | - |
| 2 | Very mild | 5mg Pred^4^  1g MMF^5^  IVIg | 65 | 640 |
| 3 | No symptoms | 7.5mg MTX^6^ | 3.5 | 80 |
| 4 | Moderate | 5mg Pred  150mg AZA^7^ | 4.6 | - |
| 5 | Mild | IVIg | - | - |
| 6 | Mild | IVIg | - | 320 |
| 7 | Moderate | Tacrolimus  IVIg | - | - |
| 8 | Very mild | 100mg AZA | 190 | - |
| 9 | No symptoms | 150mg AZA | - | - |
| 10 | Moderate | 150mg AZA | 560 | - |
| 11 | No symptoms | None | 58 | 80 |
| 12 | Very mild | 7.5mg MTX^6^ | 110 | 1280 |

^1^ daily medication at time of study

^2^ as determined by ^125^I-radio receptor assay for anti-acetylcholine receptor (AChR) IgG in nmol/L (cut-off value= 0.4 nmol/L) ^3^ and indirect immunoflourescence for anti-titin IgG (cut-off value: titer of 80).

^3^ intravenous immunoglobulins

^4^ prednisolone

^5^ mycophenolate mofetil

^6^ methotrexate (medication per week)

^7^ azathioprine
